# Diagnostic and Critical Care Challenges in Rapidly Progressive Interstitial Lung Disease Secondary to Multiple Myeloma

**DOI:** 10.7759/cureus.98122

**Published:** 2025-11-29

**Authors:** Jadav Sainath, Chinmaya K Panda, Amit Kumar, Satish Patel, Rupender Sharma

**Affiliations:** 1 Anaesthesiology, Critical Care and Pain Medicine, All India Institute of Medical Sciences, Raipur, Raipur, IND; 2 Medical Oncology, All India Institute of Medical Sciences, Raipur, Raipur, IND

**Keywords:** co-occurence, drug-induced interstitial lung disease, extramedullary multiple myeloma, hypoxic respiratory failure, interstitial lung disease, multiple myeloma, myelomatous lung involvement, rapidly progressive interstitial lung disease, type 1 respiratory faliure, usual interstitual pnuemoniae

## Abstract

The concurrent presentation of multiple myeloma (MM) and interstitial lung disease (ILD) remains exceptionally rare, with limited literature describing optimal management strategies for this dual pathology.

We report a complex case of a 62-year-old male with newly diagnosed MM with intermediate-risk cytogenetics (del13q, gain 1q) who concurrently presented with rapidly progressive ILD demonstrating a usual interstitial pneumonia (UIP) pattern. The patient's 6-month clinical course was marked by multi-organ involvement, recurrent infections, thromboembolic complications, and multiple cardiopulmonary crises culminating in a fatal outcome.

This case illustrates the profound diagnostic and therapeutic challenges when dual malignancy and progressive pulmonary fibrosis coexist, highlighting the need for aggressive multidisciplinary management, heightened awareness of infection risk, and early consideration of palliative care discussions.

## Introduction

Multiple myeloma (MM) is the second most common hematologic malignancy, characterized by the clonal proliferation of neoplastic plasma cells in the bone marrow, and accounts for approximately 10-15% of all hematologic malignancies [1]. Separately, interstitial lung disease (ILD) encompasses a heterogeneous group of diffuse parenchymal lung disorders, defined by progressive fibrosis of the lung interstitium. The etiologies of ILD are varied, including idiopathic pulmonary fibrosis (IPF), connective tissue disease-associated ILD, and iatrogenic drug-induced toxicity [2].

While both MM and ILD confer significant morbidity and mortality individually, their concurrent presentation is exceptionally rare [3]. Pulmonary involvement in MM is uncommon but can manifest in several distinct patterns, including direct parenchymal infiltration by plasma cells, the formation of solitary pulmonary plasmacytomas, or as a secondary iatrogenic complication of therapy [4-6]. When direct infiltration occurs, it can be radiographically indistinguishable from other forms of ILD or may mimic common conditions such as pneumonia or acute respiratory distress syndrome (ARDS), posing a significant diagnostic challenge [7].

The etiopathogenesis of this association remains poorly understood. Proposed mechanisms range from cytokine-mediated fibrotic pathways driven by the underlying plasma cell dyscrasia to iatrogenic lung injury from chemotherapeutic agents such as proteasome inhibitors [8]. This ambiguity makes attribution difficult and complicates therapeutic decisions. We report a case of a patient found to have coexistent multiple myeloma and ILD, adding to the limited body of literature, and highlight the diagnostic and therapeutic challenges encountered.

## Case presentation

A 62-year-old male, controlled hypertensive, presented to the medicine outpatient with lower urinary tract symptoms (dysuria, increased urinary frequency, nocturia) and nocturnal cough. He had a long-standing history of bilateral ankle pain (8 years’ duration). Initial evaluation attributed these complaints to common geriatric conditions such as urinary tract infection and suspected rheumatoid arthritis, and managed them conservatively. Two weeks later, the patient presented with upper back pain that was aggravated with postural changes. Magnetic resonance imaging of the dorsal spine revealed an acute pathologic fracture of T8 vertebra with mild-to-moderate central spinal cord compression, raising suspicion for underlying neoplastic disease. Concurrent with back pain, the patient developed chest pain and progressive dyspnea. High-resolution computed tomography of the chest demonstrated ILD with usual interstitial pneumonia (UIP) pattern, characterized by bilateral involvement with ground-glass opacities and reticular patterns. The patient was initiated on nintedanib (antifibrotic agent) and prednisolone. For back pain, he was managed conservatively.

However, one month later, he presented with multiple episodes of diarrhea, vomiting, and abdominal pain. Imaging revealed pyelonephritis. Acute kidney injury developed with elevated serum creatinine (2.64 mg/dL, normal 0.9-1.3) and urea (65 mg/dL, normal 12-42), requiring multiple hemodialysis sessions for hyperkalemia management. During that hospitalization, the patient developed hospital-acquired pneumonia (HAP) and deep vein thrombosis in the right femoral vein post-hemodialysis cannulation. Taking all the history into consideration, the patient was advised to undergo serum electrophoresis, which revealed monoclonal gammopathy with IgG and Lambda light chains. On further evaluation, bone marrow biopsy confirmed the diagnosis with findings of diffuse plasma cell involvement with both mature and immature forms (plasmablasts) in sheets, grade 0 reticulin fibrosis, and cortico-cancellous bone architecture. Cytogenetic analysis via fluorescence in situ hybridization (FISH) demonstrated del(13q) in 10% and gain 1q in 10% of interphase cells, establishing intermediate-risk disease without del(17p). Urine culture isolated Enterococcus species with a colony-forming unit (CFU) count of >105 and demonstrated sensitivity to fosfomycin. Accordingly, a 3 g oral fosfomycin sachet was administered as per standard treatment protocols. Laboratory investigations demonstrated hemoglobin 6.6 g/dL, platelet count 287,000/cumm, elevated ESR (100 mm/h), and C-reactive protein (53.70 mg/L), Calcium: 10.22 mg/dL, Albumin: 3.07 g/dL, Globulin: 4.3 g/dL (significantly elevated).

He received induction chemotherapy with cyclophosphamide, bortezomib, and dexamethasone, along with continued antifibrotic therapy (nintedanib, methylprednisolone). After resolution of acute complications (acute kidney injury (AKI), urinary tract infection (UTI), HAP), the patient received a second chemotherapy cycle and was discharged from the hospital. After three cycles of chemotherapy, repeat serum electrophoresis was negative, suggesting initial disease response. However, one week later, he presented again with fever and dysuria. Urine culture revealed Klebsiella pneumoniae. Despite outpatient antibiotic therapy, symptoms persisted, requiring admission for intravenous antibiotics. Septic features persisted, followed by dyspnea, which got aggravated over 5 days, necessitating invasive mechanical ventilation. He was transferred to the critical care unit (CCU) on vasopressor support for further management.

**Figure 1:**
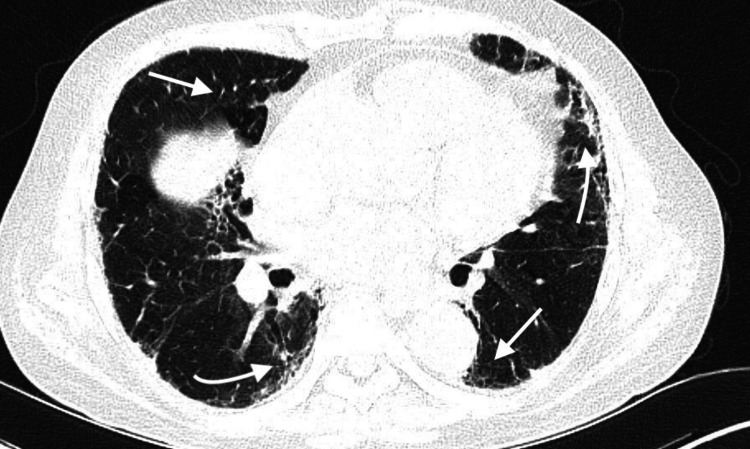
HRCT- Bilateral Inter and Intralobular septal thickening with patchy ground-glass opacities HRCT: high-resolution computed tomography

High-resolution computed tomography of the thorax demonstrated ILD progression with patchy ground-glass opacities in the right upper lobe, bilateral inter and intralobular septal thickening (Figure 1), and mediastinal lymphadenopathy (largest node 1.2 cm in the left lower paratracheal region), with possible superimposed infection. ILD workup was done by serologic evaluation, which showed a negative antinuclear antibody (ANA) test (AC-0 pattern) at a screening dilution of 1:100, with borderline anti-cyclic citrullinated peptide (anti-CCP) antibody levels (4.42 U/mL; normal <5 U/mL) and a mildly elevated rheumatoid factor (18 IU/mL; normal <15 IU/mL). Antineutrophil cytoplasmic antibodies were negative, with myeloperoxidase (MPO) and proteinase 3 (PR3) levels of 6.04 (negative ≤20) and 8.65 (negative ≤16), respectively. The hypersensitivity pneumonitis panel demonstrated markedly elevated Alternaria alternata-specific IgG (99 mgA/L; normal <30.0 mgA/L), along with raised *Aspergillus fumigatus*-specific IgG (35.50 mgA/L; normal <27.0 mgA/L) and pigeon serum protein, feather, and droppings-specific IgG (39.10 mgA/L; normal <30.0 mgA/L), while IgG levels to *Cladosporium herbarum*, *Penicillium chrysogenum*, and *Mucor racemosus* remained within reference ranges. Serum angiotensin-converting enzyme (ACE) was 44 U/L (reference range 12-68 U/L), consistent with a value within normal limits (refer to Table 1).

**Table 1:** Serological studies for ILD CCP: cyclic citrullinated peptide; ILD: interstitial lung disease

Serologic Studies	Result	Normal Value
Antinuclear antibody	Negative (AC-0)	
Myeloperoxidase (MPO)	6.04	<20
Proteinase 3 (PR3)	8.65	<16
Angiotensin-converting enzyme	44	12-68 U/L
Anti-CCP	4.42	<5 U/ml
Rheumatoid factor	18	<15 IU/ml
Hypersensitivity pneumonitis panel		
Aspergillus fumigatus-specific IgG	35.5	<27.0 mgA/L
Pigeon serum protein IgG	39.1	<30.0 mgA/L
Alternaria alternata-specific IgG 99	48.2	<30.0 mgA/L

After initial stabilization with fluid resuscitation and ventilatory optimization, the patient was extubated. However, within a week, the patient developed respiratory distress, needing re-intubation. Broad-spectrum antimicrobials were empirically initiated in view of lower respiratory tract infection. Tracheal culture developed difficult-to-treat Pseudomonas for which ceftazidime-avibactam with aztreonam was initiated. In view of recurrent clot-related blockage, tracheostomy was planned, and by the sixth day, the patient was weaned off mechanical ventilation. Lower limb Doppler ultrasound revealed near-normal thrombus in the left common femoral and great saphenous veins, with anticoagulation managed sequentially with unfractionated heparin. Clinical hypercoagulable state was correlated with the thromboelastogram (TEG) finding. Despite several successful weanings, he had developed progressive respiratory failure, probably due to worsening ILD. In view of persistent hypoxia and hypercoagulable state, acute pulmonary embolism was ruled out by CT pulmonary angiography. The course of the disease progressed rapidly over the next 2 days, leading to severe hypoxia and hypotension that eventually led to the death of the patient following unsuccessful resuscitation attempts.

## Discussion

MM is a clonal plasma cell malignancy primarily involving the bone marrow. Extramedullary MM (EMM) refers to the presence of plasma cell tumors outside the bone marrow, resulting either from direct extension of osseous lesions through cortical bone or hematogenous dissemination to soft tissues and visceral organs. The incidence of EMM ranges from approximately 0.5-4.8% at diagnosis to 3.4-14% in relapsed or refractory settings, with common sites including liver, lymph nodes, central nervous system, and, more rarely, lung parenchyma. Pulmonary involvement is exceptionally rare and clinically challenging, often mimicking infectious or malignant processes radiologically, requiring histopathological confirmation [9].

The co-occurrence of MM and ILD is exceptionally rare in the literature. While MM is associated with various pulmonary manifestations (including plasmacytoma, amyloidosis, and secondary infections), primary ILD with MM is uncommonly reported. Similarly, while ILD has numerous etiologies, malignancy-associated ILD (specifically MM-associated) is not well-established [1].

The patient’s diagnosis of MM was established through: 1) Clinical presentation with constitutional symptoms and pathologic fracture. 2) Monoclonal gammopathy (IgG/Lambda) on serum electrophoresis. 3) Increased plasma cells in bone marrow (>10%). 4) Cytogenetic abnormalities: Del(13q) and Gain 1q. 5) CRAB criteria: Calcium elevation (C) - present (10.22 mg/dL); Renal dysfunction (R) - present (Cr 2.64); Anemia (A) - present (Hb 6.6); Bone disease (B) - present (pathologic fracture) The patient met diagnostic criteria for MM and demonstrated intermediate-risk cytogenetics based on FISH findings [10].

The etiology of ILD in this patient remains multifactorial and possibly represents a unique association with MM [11]. The rarity of MM with concurrent ILD reflects limited understanding of their pathophysiologic association. Potential Mechanisms: 1) Plasma cell-derived cytokine production: Malignant plasma cells in MM produce multiple cytokines (IL-6, TNF-α, IL-1β) that may trigger pulmonary fibrosis through Th2-mediated immune responses. 2) Infectious trigger: The elevated specific IgG antibodies to multiple fungal antigens (Aspergillus fumigatus, Alternaria alternata, pigeon protein) suggest possible hypersensitivity pneumonitis as a contributing etiology. 3)Chemotherapy-related: While nintedanib is antifibrotic, bortezomib has been associated with pulmonary complications in rare cases. 4) Immunosuppression-related: The combination of MM-induced immunosuppression and intensive immunosuppressive therapy(corticosteroids, nintedanib) predisposed to recurrent infections. 5)Autoimmune component: Positive rheumatoid factor and anti-CCP suggest a possible underlying autoimmune diathesis.

The UIP pattern with rapid 6-month progression differs from classic IPF, suggesting malignancy-accelerated fibrosis. Renal dysfunction resulted from myeloma kidney (Lambda light chain precipitation), hyperuricemia, and sepsis. The patient experienced cascade infections from immunosuppression, prolonged hospitalization, and invasive procedures. Two thromboembolic events reflected malignancy-associated hypercoagulability. The patient unfortunately succumbed prior to undergoing a lung biopsy, due to the aggressive nature and poor prognosis associated with pulmonary involvement in MM. This clinical course is consistent with literature reports indicating that pulmonary extramedullary myeloma and related thoracic complications often lead to rapid clinical deterioration and high mortality, frequently before definitive histopathological confirmation can be obtained [12].

## Conclusions

This case emphasizes the profound complexity of managing MM concurrent with progressive ILD. Despite multidisciplinary care and initial chemotherapy response, progressive respiratory failure, recurrent multidrug-resistant infections, and metabolic derangements proved overwhelming. The poor prognosis when both conditions coexist and progress warrants early palliative care discussions, careful infectious disease monitoring, and potentially modified chemotherapy strategies in future similar cases.
